# Momentary Minority Stress, Nicotine Use, and Craving: Moderation by Nicotine-Use Motives Among Sexual Minority Youth

**DOI:** 10.1080/15374416.2024.2395267

**Published:** 2024-09-17

**Authors:** Wouter J. Kiekens, Jamie E. Parnes, Hayley Treloar Padovano, Robert Miranda, Ethan H. Mereish

**Affiliations:** a Department of Sociology, (ICS) Interuniversity Center for Social Science Theory and Methodology, University of Groningen; b Center for Alcohol and Addiction Studies, Brown University; c Department of Psychology, University of Maryland

## Abstract

**Objective:**

This pre-registered analysis aimed to examine the moderating role of nicotine-use motives on the association between minority stress and nicotine use and craving among sexual minority youth.

**Method:**

Data stem from a 30-day ecological momentary assessment study conducted among 83 sexual minority youth ages 15–19 years old (*M* age = 17.96, *SD* = 1.10; 56.63% cisgender women; 73.5% non-Hispanic White). Participants were instructed to complete at least four assessments per day on wireless devices. Two types of multilevel models were estimated: models predicting day-level nicotine use and models predicting momentary nicotine use craving.

**Results:**

Experiencing minority stressors was not associated with day-level nicotine use, but it was associated with greater momentary nicotine craving. Nicotine use-motives did not moderate the association between minority stress and nicotine use. In contrast, stress-reduction motives, assessed as a person-level trait, moderated the association between minority stress and nicotine craving, such that nicotine craving after experiencing a minority stressor was consistently higher relative to when minority stress had not been reported. Sensitivity analyses that examined associations between minority stress and nicotine use on a given day, regardless of temporal order, showed that minority stress was associated with higher odds of nicotine use on that day.

**Conclusions:**

Experiencing minority stressors did not predict day-level nicotine use but does contribute to greater momentary nicotine craving, informing minority stress theory. Consistency of the minority stress and nicotine craving relation, largely regardless of trait-level motives, highlights the potential context dependence of nicotine craving among sexual minority youth.

Tobacco and other forms of nicotine use (e.g., vaping; from now on referred to as nicotine use) among adolescents and young adults, herein referred to as youth, have steadily declined over the past two decades (Schulenberg et al., 2018), except for e-cigarette use (Cooper et al., 2022). This trend is significant for public health, especially considering that nearly 30% of cancer-related deaths can be attributed to tobacco use (American Cancer Society [ACS], 2022) and early tobacco use is a known risk factor for later nicotine dependence (Kendler et al., 2013). Despite this decline, sexual minority youth (e.g., lesbian, gay, and bisexual youth) remain a high-risk group for nicotine use (Fish et al., 2019; Goldbach et al., 2017; Marshal et al., 2008), with for instance 9.7% of heterosexual youth reporting past 30-day cigarette use and 13.4% to 23.1% of sexual minority youth. Not only do sexual minority youth use nicotine and other substances more often than their heterosexual peers, they also have an earlier onset of nicotine use, with odds ratios of smoking before the age of 13 ranging from 2.15 to 6.99 (Watson et al., 2018). Improving our understanding through which mechanisms (e.g., minority stress, nicotine use motives) sexual minority youth experience heightened liability for nicotine use provides more specific intervention targets for cessation interventions tailored for sexual minority youth (Fish, 2020; Mereish, 2018; Russell & Fish, 2016). This is especially relevant as youth’s nicotine use may predispose them to future nicotine use disorders and could interfere with brain development (Levy et al., 2019).

## Minority Stress and Nicotine Use and Craving

Sexual minority youth’s higher rate of nicotine use is often explained as a means of coping with minority stress (Brooks, 1981; Hatzenbuehler, 2009; Meyer, 2003). In addition to the general stress experienced by all youth, sexual minority youth face chronic minority stressors related to their sexual orientation. Such stressors include heterosexist victimization, discrimination, and family rejection (Brooks, 1981; Hatzenbuehler, 2009; Meyer, 2003). Among sexual minority youth, associations between experiencing minority stress and substance use, including nicotine use, are well documented (Goldbach et al., 2014). With, for instance, heterosexism, sexual or gender identity-related victimization, sexual or gender identity-related family rejection and violence being associated with higher nicotine use (Gamarel et al., 2020; Mereish, Fish, et al., 2022; Newcomb et al., 2014).

Research on within-person, intraindividual variability in experiencing minority stress can inform how nicotine might be used to cope with minority stress throughout the day. Mounting research has started to examine the within-person momentary associations between minority stress and nicotine use. Much of this work leverages ecological momentary assessment (EMA) methods whereby minority-stress experiences are reported throughout the day along with affect, craving, and substance use. This research has found mixed findings. Some identified associations between minority stressors and contemporaneous nicotine use among adults (Livingston et al., 2017), while others (with an initial analysis of the present study sample) found no direct, temporally sequenced association between adolescents’ minority stress experiences and subsequent nicotine use (Mereish, Treloar Padovano, et al., 2022). Thus, more research is needed to better understand the pathways and conditions under which minority stress leads to nicotine use across study samples and settings.

Although minority stress might be momentarily associated with nicotine use, youth’s access to nicotine products and the ability to use may be restricted in some settings, and this restriction may potentiate nicotine craving. It is therefore important to assess nicotine craving in addition to nicotine use. In general, drug craving, such as nicotine craving, is central to addiction (Drummond, 2001). Nicotine craving is a symptom of nicotine dependence and a predictor of relapse among youth (Colby et al., 2000), and experiencing general stressors is associated with higher nicotine craving (Buchmann et al., 2010; Childs & de Wit, 2010). Thus, one would expect that sexual minority youth who use nicotine would have higher nicotine craving on days when they experience minority stress, in line with a recent study using the same data as ours (Mereish, Treloar Padovano, et al., 2022; Parnes et al., 2023).

## Nicotine-Use Motives

Although sexual minority youth might use or crave nicotine as a response to minority stressors, there might be differences in the strength of this association. Examining nicotine-use motives could help provide a more fine-grained understanding of how nicotine use or craving is associated with minority stressors. Motives for substance use are often conceptualized as the underlying reason for substance use (Cooper et al., 2016; Cox & Klinger, 1988). Such trait-like motives are thought of as the motives that generally underlie substance use behaviors, such as nicotine use (Cooper et al., 2016). Different motives with unique precipitating factors have been distinguished for nicotine use. First, social motives reflect use to enhance positive experiences with, for instance, peers (e.g., “Smoking makes it easier to be social at a party”). Second, enhancement motives reflect use to enhance positive emotions, for instance relieving boredom to obtain an alteration in arousal (e.g., “Smoking makes me feel more energetic”). Third, conformity motives refer to use to avoid social rejection (e.g., “It helps to fit in with others”). Last, stress reduction motives reflect use to cope with negative emotions or emotional dysregulation (e.g., “It helps me to forget about worries that I have;” Piko et al., 2007; Votaw & Witkiewitz, 2021; Wills et al., 1999). A review study found that these are often reported as motives for using nicotine and are important drivers explaining nicotine use (Cooper et al., 2016). While research on nicotine-use motives among sexual minority people is scarce, scholars have indicated that sexual minority youth are motivated to use nicotine to cope with negative affect related to minority stress (Feinstein & Newcomb, 2016; Mereish, 2018; Youatt et al., 2015). Although nicotine motives, in general, are associated with nicotine use (Cooper et al., 2016), given the contributions of minority stress on nicotine use, *stress reduction or coping motives* may be especially salient for sexual minority youth.

Nicotine-use motives, such as stress reduction motives, are crucial in directly predicting nicotine use, but they might also be informative to better understand nuanced associations between minority stress and nicotine use. That is, sexual minority youth who are specifically high on stress reduction motives might be more likely to use nicotine after experiencing stressors, such as minority stress, than peers who infrequently endorse this motive for nicotine use. Similarly, as a reaction to minority stress, sexual minority youth with greater stress reduction motives may also have stronger nicotine craving than youth with lower stress reduction motives. In line with this, Black American adolescents were more likely to use substances when they experienced discrimination if they endorsed using substances as a coping mechanism (Gerrard et al., 2012). However, we are unaware of any studies that test the moderating role of stress reduction motives on the associations between minority stress and nicotine use and craving among sexual minority youth. Analysis of temporally sequenced associations in real-world settings is especially relevant given the inconsistent findings in EMA research on the association between minority stress and momentary nicotine use (Livingston et al., 2017; Mereish, Treloar Padovano, et al., 2022; Parnes et al., 2023).

## Current Study

Whether sexual minority youth use or crave nicotine as a response to minority stressors might depend on their nicotine motives, especially stress reduction motives. Therefore, we conducted secondary data analysis of EMA data and pre-registered the current study. The primary objectives of the original EMA study were to examine the mediating role of craving and affect on the momentary associations between minority stressors and subsequent nicotine use among sexual minority youth who actively used nicotine (Mereish, Treloar Padovano, et al., 2022). For the present study, we leveraged EMA data to examine how sexual minority youth’s responses to minority stressors in daily life may depend on their unique motivations for nicotine use. We hypothesized that the association between experiencing a minority stressors and subsequent nicotine use and nicotine craving would be stronger for sexual minority youth with high levels of stress reduction motives than for those with low levels of stress reduction nicotine-use motives. We anticipated that stress reduction motives would play a unique and specific role in understanding the real-world effects of stress experiences on sexual minority youth. As such, we hypothesized that social, self-enhancement, and boredom-relief motives would not moderate the associations between minority stress and nicotine use and craving. The novelty of the present study lies in the focus on how nicotine-use motives may moderate the association between experiencing minority stressors and subsequent nicotine use *and* nicotine craving and differentiate the present study from recent studies using the same data (Mereish, Treloar Padovano, et al., 2022; Parnes et al., 2023).

## Methods

### Participants and Procedure

Data stem from an EMA study conducted among sexual minority youth (Mereish, Treloar Padovano, et al., 2022). Participants were recruited from 2018 to 2019 through social media platforms (e.g., Instagram), sexual minority youth school- and community-based organizations, sexual minority-based events (e.g., Pride), and public venues (e.g., coffee shops) in a metropolitan city in the Northeast of the U.S. Interested youth were screened for inclusion criteria over the phone and, when potentially eligible, invited to an in-person screening. Inclusion criteria were sexual minority self-identification (i.e., “How would you describe your sexual identity?”); ages 15 to 19 years old; an active consumer of any nicotine product defined by Mayhew et al. (2000) stages of smoking adolescent onset (e.g., smoking four or more cigarettes or vape/e-cigarette sessions in the past 7 days), assessed by a Timeline Followback Interview, and breath carbon monoxide readings; experienced at least three sexual orientation-based minority stressors in the past 30 days (Mereish et al., 2021); and able to read English. Exclusion criteria were involvement in smoking cessation treatment (including the use of nicotine replacement products), psychosis, or suicidality. In total, 237 youth were phone screened, and 90 youth were invited for in-depth in-person screenings. Of those, 85 were enrolled as participants. Youth ages 18 or 19 years provided informed consent. Those younger than 18 years provided assent; permission was obtained from their parent/guardian.

Eligible youth completed a baseline survey and received training for the EMA surveys. Participants were asked to complete 30 days of EMA and return to the lab for an end-of-study visit after this period. Participants were compensated with gift cards for their time. When needed, travel expenses for these visits were compensated. Participants were asked to complete EMA on their personal wireless devices; if they did not own such a device, they received one for study purposes. EMA reports were completed through MetricWire Inc. (Ontario, Canada).

To capture nicotine use, nicotine craving, and minority stress, we culled data from two types of EMA reports: random reports and nicotine reports. Random reports assessed nicotine use and minority stress since the last report and nicotine craving in the present moment. They were randomly delivered five times a day from 9 am to 11:15 pm with a minimum of 2.75 h between each random report. These had to be completed within 30 min of notification, and reminders were sent (push notifications 10 and 20 min after the initial prompt and a text message 5 min before expiration) until the report was completed or expired. Our EMA program did not allow participants to “delay” or “suspend” prompts if currently unable to reply (e.g., driving, in class). Participants were considered fully compliant if they completed at least four random reports per day. Participants were also instructed to self-initiate nicotine reports immediately before any nicotine use, which captured information about the quantity and type of nicotine use. During random reports, participants were asked if they had used nicotine recently, and if so, they were directed to complete a nicotine report. All reports were time-stamped.

Participants were compensated $10 for the in-person screening, and, if eligible, an additional $35 for the baseline visit (total $45) and $30 for the end-of-study visit. In addition, participants were compensated up to $4.50 daily based on their daily responses ($1 for morning assessments, $0.50 for each of the five daily device-initiated random assessments, and $1 for bedtime assessments). Additional “bonuses” of $10 were offered for every 10-day period with a compliance rate of >80% for random prompts. Thus, the total possible amount participants can earn over the course of the study was $245. The Institutional Review Board of the last four authors approved the study protocol.

### Measures

#### Baseline Measures

##### Nicotine-use motives.

Nicotine-use motives were assessed during the baseline survey at the first lab visit using the Tobacco Motives Scale (TMS; Wills et al., 1999, 2002). The TMS consists of four subscales assessing the following nicotine use subscales: stress reduction (five items, α = .86, example item: “Smoking helps you forget about worries”), social (four items, α = .85, example item: “Smoking helps you fit in with other people”), self enhancement (four items, α = .72, example item: “Smoking makes you feel more energetic”), and boredom relief (two items, α = .84, example item: “You can smoke when there’s nothing better to do”). Additionally, for this study, we developed two exploratory items that measured LGBTQ-specific nicotine-use motives (i.e., “Smoking helps you deal with stress related to your LGBTQ identity,” [LGBTQ-specific stress reduction motives] “Smoking helps you fit in with other LGBTQ people” [LGBTQ-specific social motives]). Answer options for all questions ranged from 1 = *Not at all true* to 5 = *Very true*. We calculated an average for each TMS subscale (see Table 2), while LGBTQ-specific motives were analyzed individually in supplemental analyses.

##### Covariates.

Time-invariant covariates measured at baseline included participant age (years), gender identity (0 = *Cisgender male*, 1 = *Cisgender female*, 2 = *Gender minority;* dummy coded), sexual orientation (0 = *Gay/lesbian*, 1 = *Bisexual*, 2 = *Pansexual or queer*; dummy coded), race (0 = *White*, 1 = *Racial minority*), and ethnicity (0 = *Hispanic/Latine*, 1 = *non-Hispanic/Latine*). Additionally, to estimate socioeconomic status, we assessed whether participants reported receiving free or reduced-cost school lunch or not (0 = *No*, 1 = *Yes*) as an indicator of socioeconomic disadvantage. Lastly, to assess baseline mental health symptoms, we administered the Social Anxiety Scale for Adolescents (SAS-A; la Greca & Lopez, 1998) and the Center for Epidemiologic Studies Depression Scale (CES-D; Radloff, 1977). The SAS-A measures the frequency of 20 social anxiety symptoms (α = .94, example item: “I feel that others are making fun of me.”) on a 5-point scale from 1 = *Not at all* to 5 = *All the time*. The CES-D measures the frequency of 20 depression symptoms (α = .90; example item: “I felt sad.”) on a 4-point scale from 1 = *Rarely or none of the time* to 5 = *Most or all the time*. Both the SAS-A and CES-D are scored by calculating a total sum score.

#### EMA Measures

##### Day-level nicotine use.

On random reports, participants were asked “What type(s) of nicotine or tobacco did you use since your last TED report?.” Answer options were *Cigarettes, Vapes, E-cigs, Cigars, Chewing tobacco, snuff, or dip, Hookah or waterpipe, Other Nicotine/Tobacco product, None*. On nicotine use reports, participants were asked “Have you started using nicotine yet?” with answer options *Yes* and *No*. Nicotine use was coded based on if the participant endorsed any nicotine use via random and/or nicotine use reports that day (0 = *No*, 1 = *Yes*).

##### Momentary nicotine craving.

To measure nicotine craving, participants were asked during the random reports, “How strong is your urge to use nicotine right now?” Answers were selected on a sliding bar, with response options from 0 = *No urge* to 10 = *Strongest ever* (Tiffany, 2010).

##### Momentary minority stress.

To align with study inclusion criteria and prior literature using scale-based assessments of individual differences in minority stress, we used the Everyday Identity Stress Scale (EISS) to measure momentary minority stress (Mereish et al., 2021). The EISS has been validated to measure minority stress among sexual minority youth in daily diary studies. It consists of nine items describing a minority stressor that might have occurred during the day. Questions were adapted to reflect whether a minority stressor occurred since the previous random report. Example questions reads “Have you been targeted or harassed because of your identity since your last TED [The Electronic Diary] report?” and “Were you ignored or isolated or made to feel invisible since your last TED report?.” Youth indicated whether any of the nine stressors commonly experienced by individuals with minoritized identities occurred since their last random report, coded as 0 = *No event* and 1 = *Any event*. Consistent with Mereish et al. (2021) and the measure’s focus on intersecting minority stressors, we coded any endorsed stressor as a minority stressor. For day-level analyses, minority stress was aggregated to the day level (0 = *No stressors that day prior to nicotine use*, 1 = *Any stressors that day prior to nicotine use*).

##### Covariates.

We tested several momentary time-varying covariates. Timestamps from random reports were used to calculate the time of day (0 = *6pm–12am*, 1 = *12am–6am*, 2 = *6am–12pm*, 3 = *12pm–6pm*). Additionally, during random reports, participants were asked where they were located (home, friend’s home, dorm, school, work, club/bar, restaurant, vehicle, another public place, or another private place), which was coded as 0 = *Elsewhere* or 1 = *Home/dorm*. Random reports also assessed who the participant was with (no one, friends, boy/girlfriend, parent(s), other family member(s), other relative(s), coworker(s), or someone else). To control for peer influence on nicotine use (Abadi et al., 2022), peer presence was coded as 0 = *No peer(s)/romantic partner present*, 1 = *Peer(s)/romantic partner present* and we controlled for recent nicotine use. A day-level time-varying covariate was weekend status (0 = *Monday to Thursday*, 1 = *Friday to Sunday*).

### Analytical Strategy

Study hypotheses were tested using multilevel modeling in SAS 9.4 (SAS Institute Inc, 2013), which accounts for the nested data structure (i.e., days/moments [level 1] nested within participants [level 2]). Our EMA program required participants to complete all items on random and nicotine reports, and we did not have any incomplete reports or missing EMA data. Due to software issues, we were missing current craving on 16 random reports. Minority stress theory suggests nicotine use should occur following, and in response to, minority stressors. To establish this temporal structure in the data for models predicting nicotine use, random reports completed prior to participants’ first nicotine use episode of the day were maintained for analyses, while random reports completed after nicotine use that day were excluded. In other words, any minority stressor(s) occurred *prior* to any nicotine use each day was used to predict if a participant used nicotine *after* those stressors (i.e., lagged effects) later that same day. We refer to this aggregated, day-level sample as the *day-level sample*. For nicotine craving, momentary random reports assessed minority stressors that occurred *prior* to the random report and *current* craving while completing the report, which inherently established a temporal sequence. We refer to this sample as the *momentary sample*.

The odds of day-level nicotine use were modeled using logistic regression, and momentary nicotine craving was modeled using negative binomial regression. Minority stressors were recoded for inclusion as a binary indicator of whether a stressor occurred (day-level [odds of use model] or momentary [craving model] predictor) and aggregated, person-level indicator of the proportion of days for which a minority stressor was endorsed for that person, overall (individual-difference predictor). To test for moderation, each nicotine use motive (grand-mean centered) was included along with a cross-level interaction with momentary/day-level minority stress. We calculated intraclass correlation coefficients (ICCs), a ratio of the partitioned between – and within-person participants, for odds of using nicotine using an intercept-only model in SAS. For momentary nicotine craving, an ICC was calculated in R 2022.12.0 (R Core Team, 2022) using the iccCounts package (Carrasco, 2022) to account for the variable’s negative binomial count distribution.

When building models, we initially tested multivariate associations between putative covariates and each outcome, then removed non-significant covariates. We then added momentary minority stress, nicotine-use motives (except LGBTQ-specific motives; see Supplemental Analysis), and cross-level interactions to the model to test for moderation. Random slope effects were excluded to support convergence and reduce model complexity. Parameter estimates for logistic and negative binomial models were exponentiated to calculate adjusted odds ratios (AOR) and incidence rate ratios (IRR), respectively. Significant interactions were plotted as the predicted probability of day-level nicotine use and momentary nicotine craving (Dawson, 2013).

#### Sensitivity Analyses

While modeling all motives and cross-level interactions simultaneously provides a stringent test of the unique influence of each motive, the covariance between the different motives could lead to biased results. Therefore, we also evaluated individual models for each nicotine motive as a sensitivity analysis and used a Bonferroni correction to adjust for multiple comparisons (four motives, *p* < .01).

We preregistered only examining random reports completed prior to nicotine use that day for the day-level nicotine use model. However, this ultimately excluded eight participants and 56.6% of study days, as many participants’ first report of the day was a nicotine use report. In turn, we conducted a supplementary, unregistered sensitivity analysis examining associations between minority stress and nicotine use on a given day, regardless of temporal order (using *p* < .01 to correct for multiple comparisons). Lastly, during the review process, we tested sensitivity analyses including post-hoc covariates of baseline social anxiety and depression symptoms.

#### Supplemental Analyses

To evaluate the impact of LGBTQ-specific motives, we conducted supplemental analyses examining the moderating role of the two assessed LGBTQ-specific motives. We estimated three models, two for nicotine use and one for nicotine craving, using the same approach as our primary and sensitivity analyses (using *p* < .01 to account for exploratory analysis).

## Results

After removing two participants who only completed 1 day of EMA and reports missing craving data, the *momentary sample* (in which nicotine craving will be assessed) included *N* = 83 participants who completed 5,897 random reports over 2,010 study days. Participants completed EMA reports on an average of 24.22 days (SD = 7.59, range 3–30). About 79.5% of participants completed EMA reports on at least 80% of the days they were in the study (mean compliance = 88.2%). On days when participants completed EMA reports, they completed an average of 2.75 random reports (SD = 0.93, 68.8% compliance). EMA random report compliance was not associated with age (*r* = .04, *p* = .72), gender identity (*F*(1) = 0.27, *p* = .61), sexual orientation (*F*(1) = 0.44, *p* = .51), or total number of random reports endorsing minority stress (*r* = .06, *p* = .58). The *momentary sample* had a mean age of 17.96 (SD = 1.10, range 15–19), were 56.6% cisgender women (25.3% cisgender men, 18.1% gender minority), 84.3% White, and 18.1% Hispanic/Latine. Overall, participants reported nicotine use on 76.2% of study days, minority stress on 27.9% of study days, and minority stress on 12.6% of random reports.

The *day-level sample* (in which nicotine use will be assessed) included 75 participants with a total of 872 study days (i.e., 1,138 days were excluded because nicotine use was the first report of the day; but see also supplemental models including all study days). This sample had a mean age of 18.0 (SD = 1.11, range 15–19), were 58.7% cisgender women (25.3% cisgender men, 16.0% gender minority), 84.0% White, and 17.3% Hispanic/Latine. In this sample, participants reported nicotine use on 45.5% of study days and minority stress on 16.3% of study days. See Table 1 for full participant demographic information and Table 2 for focal variables’ descriptive information.

### Multilevel Models

First, we focus on the analyses in the *day-level sample* in which day-level nicotine use was the outcome variable. Here, only weekend day was associated with the odds of day-level nicotine use and thus included as covariate. The estimated ICCs suggested that 24% of the variance in day-level nicotine was associated with between-person factors. Nicotine motives, day-level minority stress, and the cross-level interactions were not associated with odds of day-level nicotine use (see Table 3).

**Figure 1.**
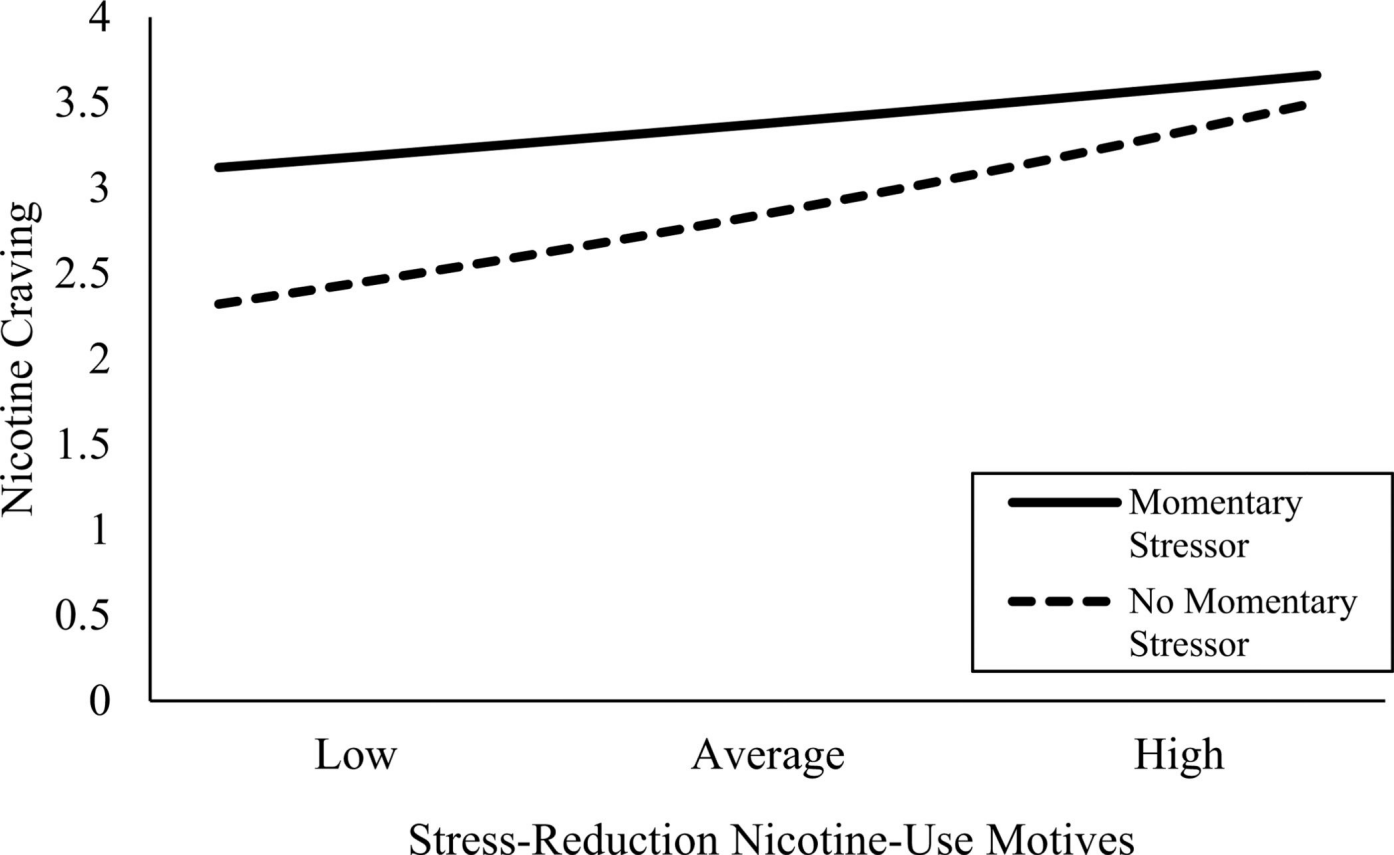
Interaction plot for minority stress and stress reduction nicotine use motives predicting nicotine craving. Craving was estimated using reference groups for location (not at home/dorm) and recent nicotine use (none).

Second, we focus on the analyses in the *momentary sample* with nicotine craving as outcome variable. Location and recent nicotine use were associated with momentary nicotine craving and therefore included as covariates. The ICCs suggested that 72% of the variance in momentary nicotine craving was associated with between-person factors. Momentary minority stress (IRR = 1.15, *p* < .0001), person-level minority stress (IRR = 4.69, *p* = .02), and the cross-level interaction with stress reduction motives (IRR = 0.94, *p* = .04) were significantly associated with greater momentary nicotine craving (see Table 3). Probing the interaction revealed that nicotine craving was consistently higher following minority stress experiences, and this effect appeared consistent across levels of stress reduction motives. In the absence of a recent minority stress experience, however, participants’ stress reduction motives were positively associated with greater nicotine craving (see Figure 1). No other nicotine motives or cross-level interactions were associated with momentary nicotine craving.

### Sensitivity Analyses

First, we individually modeled each nicotine use motive as a sensitivity analysis to account for covariance between the different nicotine-use motives. From the models in the *day-level sample* examining day-level nicotine use, weekend was the only covariate associated with odds of use, while minority stress, nicotine-use motives, and the cross-level interactions were all unassociated with the odds of nicotine use. Across all models in the *momentary sample*, assessing momentary nicotine craving, momentary minority stress (IRR range 1.13 to 1.16, *p* < .0001) and person-level minority stress (IRR range 5.42 to 8.24, *p* range .001 to .01) were consistently associated with greater momentary nicotine craving (see Table S1 in the Supplementary Material). Additionally, there was a significant cross-level interaction with stress reduction motives (IRR = 0.94, *p* = .01). Similar to the main analyses, the association between momentary minority stress and craving attenuated as stress reduction motives (see Figure S1 in the Supplementary Material).

Next, we conducted supplementary sensitivity analyses examining associations between any minority stress and odds of nicotine use on a day (ICC=.46 in these data), regardless of temporal order. Weekend was the only covariate associated with odds of nicotine use and included as covariate. In models in which all nicotine motives variables and interactions were entered simultaneously, minority stress (AOR = 1.69, *p* = .003) was associated with greater odds of nicotine use on that day (see Table S2). When each nicotine use motive was individually modeled, minority stress was consistently associated with greater odds of using nicotine that day (AOR range 1.55 to 1.65, *p* range .004 to .009). Person-level minority stress, nicotine-use motives, and cross-level interactions were not significantly associated with odds of nicotine use (see Table S2). Lastly, sensitivity analyses controlling for post-hoc covariates of social anxiety and depression were consistent with the primary model results (see Table S3).

### Supplemental Analyses

The moderating role of two LGBTQ-specific motives (i.e., LGBTQ-specific stress reduction motives and LGBTQ-specific social motives) on the association between minority stress and nicotine use and nicotine craving. First, in the *day-level sample* with day-level nicotine use as outcome variable, no significant main or interaction effects with LGBTQ-specific motives were found. Second, in the *momentary sample* with nicotine-use craving as outcome variable, there was a significant cross-level interaction with LGBTQ-specific stress reduction motives (IRR = 0.95, *p* = .005). The association between momentary minority stress and craving attenuated as LGBTQ-specific stress reduction motives (see Figure S2 in the supplementary material) increased. Last, in analyses examining associations between any minority stress and odds of nicotine use on a day, no significant main or interaction effects with LGBTQ-specific motives were found.

## Discussion

Nicotine-use motives, assessed as person-level traits, may play a role in real-time associations between minority stress and nicotine use or craving among sexual minority youth. In secondary analyses, we utilized temporally sequenced data from moments when youth reported experiencing minority stressors and subsequent nicotine use or craving. We expected that the relations of minority stress with nicotine use and craving would be enhanced for youth who hold the belief that they use nicotine to reduce their stress, relative to youth who do not hold this belief or hold it less strongly.

Focusing on nicotine use first, temporally sequenced models did not find an association between minority stress and nicotine use. While necessary to establish a causal sequence and avoid confounding factors, inclusion of only the first nicotine use event of a given day reduced the available nicotine use events by half. The latency from waking to the first nicotine use of the day can be a function of nicotine dependence (Branstetter et al., 2020). Given that in our sample initial nicotine use occurred, on average, around 8:42am (SD = 3.89 h), initial nicotine use for the day may have occurred prior to experiencing minority stress, although one could also experience minority stressors early on the day via, for instance, social media. As excluding subsequent nicotine-use reports that day may thus not adequately represent the nature of nicotine use among those who use nicotine products who have progressed further into nicotine dependence. Alternatively, mechanisms in combination with minority stress may explain nicotine use among sexual minority youth. For example, more permissive tobacco use norms among sexual minority populations could explain their tobacco use (Boyle et al., 2020). Sexual minority youth may have more frequent exposure to tobacco marketing than heterosexual youth (Soneji et al., 2019) which has been associated with nicotine use (Emory et al., 2019).

Sensitivity analyses that examined associations between minority stress and nicotine use on a given day, regardless of temporal order, showed that minority stress was associated with higher odds of nicotine use on that day. These contemporaneous effects are consistent with the minority stress framework (Brooks, 1981; Hatzenbuehler, 2009; Meyer, 2003) and support prior literature (Livingston et al., 2017). Data exclusions, while necessary to establish temporal precedence, reduced the nicotine-use events available for analysis. Differences in selection and timing of observations may help explain discrepancies in the literature, including null effects in our primary report for this study sample (Mereish, Treloar Padovano, et al., 2022).

Contrary to our hypotheses, associations between real-world minority stress and subsequent nicotine use were not moderated by individual differences in stress reduction nicotine-use motives, nor any other nicotine-use motive. In the sensitivity analyses that examined associations between minority stress and nicotine use on a given day, regardless of temporal order, moderating effects of motives remained nonsignificant. Research has pointed to how motives inconsistently influence the association between stressors and momentary substance use, such as nicotine use (Votaw & Witkiewitz, 2021). Examination of indicators of high reactivity to negative affect, such as distress intolerance and anxiety sensitivity, could further explain inconsistent findings. That is, these indicators are pointed to as important antecedents to motives as they are associated with, for instance, coping and conformity motives for substance use (Demartini & Carey, 2010; Zvolensky et al., 2009). As such, they could potentially clarify inconsistent findings regarding the moderating role of nicotine-use motives (Votaw & Witkiewitz, 2021). That is, they might act as moderators themselves or could illuminate for whom (e.g., high or low on distress intolerance) nicotine-use motives act as moderators for the association between minority stress and nicotine use or craving.

Moving to nicotine craving, in line with our hypotheses, momentary minority stress was associated with higher momentary nicotine craving. This is in line with research demonstrating that experiencing stressors is associated with higher nicotine craving (Buchmann et al., 2010; Childs & de Wit, 2010) and our prior work with the same sample of sexual minority youth (Mereish, Treloar Padovano, et al., 2022). Building on our prior work, we found that stress reduction motives moderated the association between momentary minority stress and nicotine craving. This finding was replicated in sensitivity analyses where each nicotine use motive was individually modeled. Among our present sample of active users of nicotine products, it appears that youth with higher stress reduction motives are often experiencing low to moderate nicotine craving, suggesting there may be a multitude of triggers that induce craving for these youth. In turn, experiencing regular craving may obscure the acute impact of a minority stressor. Conversely, when someone did not experience a minority stressor, nicotine craving levels are relatively lower, and differences in trait-level stress reduction motives may have more relevance for nicotine craving levels.

Although not the specific focus of the present paper, we found few associations between nicotine-use motives and day-level nicotine use and momentary nicotine craving. That is, only in the sensitivity analyses where each nicotine use motive was individually modeled, stress reduction motives and boredom relief motives were associated with greater odds of day-level nicotine use. For momentary nicotine craving, no associations with other nicotine-use motives were found. This lack of associations contradicts the motivational model of substance use (Cooper et al., 2016). However, positive reinforcement and automatic processes (e.g., habit) have been identified as the primary motivations underlying day-level nicotine use (Votaw & Witkiewitz, 2021), and the motivational model might thus be able to only partly explain day-level nicotine use or craving. The lack of associations between nicotine-use motives and nicotine use and craving in our study is thus in line with these findings, especially since our sample reported frequent nicotine use. This calls for exploring how positive reinforcement and automatic processes might play a (moderating) role in sexual minority youth’s nicotine use and craving. Further, in our study motives were only assessed at baseline and were thus viewed as a trait of an individual. To better understand how motivations of nicotine use affect nicotine use and craving among sexual minority youth, research could instead approach motivations as momentary processes in daily life (Votaw & Witkiewitz, 2021).

Our analysis informs the growing body of literature on minority stress in sexual minority youth in two significant ways. First, we address the mixed results regarding the association between minority stress and momentary nicotine use. We found only a significant association between minority stress and odds of nicotine use on a day without taking temporal order into account. It would be an important next step to create a study design with high statistical power while also allowing to test for temporal order between minority stress and nicotine use. Second, we examined the role of nicotine use motivations. This is relevant as research on nicotine use motives among sexual minority youth is scarce *and* could illuminate heterogeneity in how minority stressors are associated with nicotine use and motives. Given that we found limited significant moderating effects of trait-level motives, our research suggest other moderators of this association should be considered in future research. One potential next step could be to better understand the role of LGBTQ-specific nicotine use motives. We examined the role of LGBTQ-specific stress reduction and social motives and found that the association between momentary minority stress and nicotine craving attenuated as LGBTQ-specific stress reduction motives increased. Further development of these items into psychometrically validated scales could provide specificity on how nicotine motives directly affect nicotine use and cravings and act as moderators.

Our study also shows that experiencing minority stressors is common among sexual minority youth (27.9% of the participated days). In general, considering how minority stress may explain sexual orientation-based health disparities, our findings underline the relevance of minimizing the exposure to minority stressors in social environments that sexual minority youth traverse. Further, results indicate that momentary minority stressors were especially associated with momentary craving to use nicotine. These results take a step toward tailoring cessation interventions for sexual minority youth. Clinicians may aim to work with sexual minority youth to identify triggers and prevent nicotine-use cravings resulting from experiences of minority stress. Future work with larger samples and greater granularity for the type of minority stressor experienced may better inform clinical intervention.

### Limitations and Future Directions

The current analysis of secondary data should be interpreted considering some limitations. First, one should be careful with generalizing the present findings. Participants were, for instance, sampled from a metropolitan city in the Northeast of the U.S., and there was an overrepresentation of youth who identified as White. Additionally, inclusion criteria required participants to report at least three sexual orientation-based minority stressors in the past 30 days. While this criterion helps ensure sufficient variability in minority stress reporting during the EMA monitoring period, it also limits the generalizability of the results to sexual minority youth who experience multiple minority stressors on a monthly basis. Second, multiple aspects of the research design influenced the compliance rates in our study, although compliance rates were comparable to other studies with substance using samples (Jones et al., 2019). Like many other EMA studies, the app used for this study did not provide the option of delaying or suspending random reports when unable to respond. Having “competing activities” is a common reason for missing EMA assessments (Fred Wen et al., 2017); however, we were not able to capture these factors in our study. Third, although we tried to establish temporal order (minority stressors preceding nicotine use or craving), the balance of strengths and limitations precludes conclusions of causation. A wider breadth of mixed-methods research is needed, which might be especially relevant to study the nature of nicotine craving and content validity of motives assessments among sexual minority youth. Our results are consistent with prior experimental work, however, that found that minority stress elicits alcohol craving among sexual minority young adults (Mereish & Miranda, 2019). Fourth, to achieve temporal order and assure minority stressors preceded nicotine use, we removed days where participants first report included nicotine use. However, the first cigarette of the day strongly predicts nicotine dependence (Branstetter et al., 2020), especially in countries such as the U.S. (Fagerström, 2003). Thus, by removing days where the first report included nicotine, we likely omitted participants with high nicotine dependence, which could have influenced our results. Fifth, momentary minority stress was considered as a binary variable, which inhibits us from drawing any conclusion on the extent of experiencing multiple minority stress and how this is associated with day-level nicotine use and momentary nicotine craving. Similarly, it was only assessed whether participants used nicotine in the surveys included in the EMA protocol, not the amount of, for instance, cigarettes they used. It was therefore not possible to examine rates of nicotine use.

Despite that the EISS assessed multiple and intersecting dimensions of minority stress, we did not assess how the studied associations differed based on race/ethnicity, gender, or sex assigned at birth. Doing so is important as interlocking systems of oppressions can influence youth’s experience with minority stressors and, ultimately, how this affects nicotine use and craving. Future research with larger sample sizes and diversity could take such an intersectional approach.

## Conclusion

The present study examined the moderating role of nicotine-use motives on the association between minority stress and nicotine use and craving. Our results found that experiencing minority stressors was, to some extent, associated with day-level nicotine use, and it was associated with greater momentary nicotine craving. Trait-level motives did not moderate the association between minority stress and daily nicotine use, and most motives did not influence the relation of momentary minority stress to subsequent nicotine craving. In specific, however, stress reduction motives moderated the momentary association between minority stress and nicotine craving, such that nicotine craving after experiencing a minority stressor was consistently higher relative to when a minority stress had not been reported. Our findings add to a growing body of work informing minority stress models of contextual influences on nicotine use among sexual minority youth. Work is underway to improve prevention and cessation interventions, which should focus on reducing minority stress at the structural, individual, and contextual levels. Sexual minority youth who are active users of nicotine products may have contextually driven motives for use and need help to manage cravings to use within a context of minority stress.

## Supplementary Material

Supplementary Material

## Figures and Tables

**Table 1.** Participant demographic characteristics.

	Momentary Sample (*N* = 83)		Day-Level Sample (*N* = 75)	
	*M*	*SD*	*M*	*SD*
Age	17.96	1.10	17.96	1.11
	*N*	*%*	*N*	*%*
Gender				
Cisgender Men	21	25.30	19	25.33
Cisgender Women	47	56.63	44	58.67
Gender Minority	15	18.07	12	16.00
Sexual Identity				
Gay or Lesbian	15	18.07	14	18.67
Bisexual	47	56.63	44	58.67
Pansexual or Queer	21	25.30	17	22.67
Race				
American IndianAlaska Native	1	1.20	1	1.33
Asian	2	2.41	2	2.67
Black/African American	3	3.61	3	4.00
White	70	84.34	63	84.00
Multiracial	7	8.43	6	8.00
Hispanic/Latino(a)	15	18.07	13	17.33

**Table 2.** Study variable descriptive statistics.

	Momentary Sample (*N* = 83)			Day-Level Sample (*N* = 75)		
	*M*	*SD*	*a*	*M*	*SD*	*a*

Nicotine-Use Motives						
Stress Reduction	3.55	1.04	.86	3.53	1.03	.85
Social	2.60	0.99	.85	2.63	0.97	.84
Self-Enhancement	2.51	0.96	.72	2.50	0.96	.72
Boredom Relief	3.49	1.23	.84	3.48	1.21	.84
LGBTQ-Specific Stress Reduction	2.96	1.30	–	3.00	1.30	–
LGBTQ-Specific Social	2.29	1.23	–	2.29	1.23	–
Person-Level Minority Stress Days^a^	0.28	0.22	–	0.15	0.20	–
Person-Level Minority Stress Reports	0.14	0.14	–	–	–	–

Nicotine Craving (grand mean)	*M (SD)* 3.91 (2.32)					

	*N*		% of days (2,010)	*N*		% of days (872)
Nicotine Use	1,532		76.22	397		45.52
Minority Stress	561		27.91	142		16.28
Weekend (Friday-Sunday)	868		43.18	360		41.28
	*N*		% of reports (5,897)			
Minority Stress	744		12.62			
Time of Day						
12am-6am	10		0.17			
6am-12pm	1,013		17.18			
12pm-6pm	2,589		43.90			
6pm-12am	2,285		38.75			
Location – Home/Dorm	3,024		51.28			
Peers Present	2,518		42.70			
Recent Nicotine Use	2,811		47.67			

a This reflects the proportion of days/reports endorsing a minority stressor.

**Table 3.** Multilevel models predicting odds of day-level nicotine use and momentary nicotine craving.

	Nicotine Use^a^			Nicotine Craving^b^		
Variable	*AOR* (95% CI)	*SE*	*p*	*IRR* (95% CI)	*SE*	*p*
Intercept	0.92 (0.66, 1.29)	0.17	.62	**3.00 (2.54, 3.55)**	**0.08**	**<.001**
*Level 1 (Within-Participants)*						
Minority Stress	0.80 (0.50, 1.27)	0.23	.34	**1.15 (1.09, 1.21)**	**0.03**	**<.001**
Location	-	-	-	**0.91 (0.88, 0.95)**	**0.02**	**<.001**
Recent Nicotine Use	-	-	-	**1.05 (1.01, 1.10)**	**0.02**	.**02**
Weekend	**1.52 (1.12, 2.06)**	**0.15**	.**008**	-	-	-
*Level 2 (Between-Participants)*						
Stress Reduction Motives	1.16 (0.81, 1.67)	0.18	.13	1.11 (0.90, 1.36)	0.10	.50
Social Motives	0.87 (0.59, 1.29)	0.20	.78	0.83 (0.66, 1.03)	0.10	.06
Self-Enhancement Motives	0.94 (0.62, 1.43)	0.21	.74	1.21 (0.96, 1.51)	0.11	.08
Boredom Relief Motives	1.28 (0.94, 1.74)	0.16	.19	1.02 (0.86, 1.20)	0.08	.87
Minority Stress	0.21 (0.03, 1.40)	0.94	.11	**4.69 (1.26, 17.46)**	**0.65**	.**02**
*Cross-Level Interactions*						
Stress Reduction Motives x Minority Stress	1.39 (0.82, 2.35)	0.27	.23	**0.94 (0.88, 0.99)**	**0.03**	.**04**
Social Motives x Minority Stress	1.17 (0.67, 2.04)	0.28	.57	0.96 (0.89, 1.03)	0.03	.27
Self-Enhancement Motives x Minority Stress	0.96 (0.49, 1.90)	0.35	.91	1.03 (0.96, 1.10)	0.03	.41
Boredom Relief Motives x Minority Stress	0.95 (0.63, 1.44)	0.21	.82	0.99 (0.95, 1.04)	0.02	.71

*Note*: Adjusted odds ratio (AOR), incidence rate ratio (IRR), and 95% confidence interval (CI). Covariate reference groups: location is elsewhere (i.e., not home/dorm), recent nicotine use is no, and weekend is weekday. Significant results are bolded.

a These analyses were conducted in the day-level sample (*N* = 75).

b These analyses were conducted in the momentary sample (*N* = 83).

## Data Availability

The data that support the findings of this study are available from the corresponding author, E. Mereish, upon reasonable request.
