# Supplementary material for: Momentary Minority Stress, Nicotine Use, and Craving: Moderation by Nicotine-Use Motives Among Sexual Minority Youth
Source: J Clin Child Adolesc Psychol. Author manuscript; Available in PMC 2026 Feb 14. (PMC11911245; doi:10.1080/15374416.2024.2395267)
Supplement: Supplementary Material [file NIHMS2023622-supplement-Supplementary_Material.docx]

| Table S1 | | | | | | | |
| --- | --- | --- | --- | --- | --- | --- | --- |
| Sensitivity Analysis – Separate Multilevel Models for each Nicotine Use Motive Predicting Odds of Nicotine Use and Nicotine Craving | | | | | | | |
| **Variable** | **Nicotine Use^1^** | | |  | **Nicotine Craving^2^** | | |
|  | ***AOR***  **(95% CI)** | ***SE*** | ***p*** |  | ***IRR***  **(95% CI)** | ***SE*** | ***p*** |
| *Stress Reduction Motives Model* |  |  |  |  |  |  |  |
| Intercept | 0.92  (0.66, 1.27) | 0.17 | .59 |  | **2.99**  **(2.52, 3.56)** | **0.09** | **<.001** |
| Minority Stress (level 1) | 0.80  (0.51, 1.27) | 0.23 | .34 |  | **1.16**  **(1.10, 1.21)** | **0.03** | **<.001** |
| Location (level 1) | - | - | - |  | **0.91**  **(0.88, 0.95)** | **0.02** | **<.001** |
| Recent Nicotine Use (level 1) | - | - | - |  | 1.05  (1.01, 1.10) | 0.02 | .02 |
| Weekend (level 1) | **1.52**  **(1.12, 2.05)** | **0.15** | **.008** |  | - | - | - |
| Minority Stress (level 2) | 0.20  (0.03, 1.28) | 0.92 | .09 |  | **5.45**  **(1.46, 20.24)** | **0.66** | **.01** |
| Stress Reduction Motives | 1.27  (0.95, 1.71) | 0.15 | .02 |  | 1.18  (0.99, 1.41) | 0.09 | .06 |
| Stress Reduction Motives x  Minority Stress | 1.36  (0.89, 2.09) | 0.22 | .15 |  | **0.94**  **(0.90, 0.98)** | **0.02** | **.009** |
| *Social Motives Model* |  |  |  |  |  |  |  |
| Intercept | 0.90  (0.64, 1.27) | 0.17 | .54 |  | **3.00**  **(2.51, 3.57)** | **0.09** | **<.001** |
| Minority Stress (level 1) | 0.79  (0.50, 1.24) | 0.22 | .30 |  | **1.13**  **(1.08, 1.18)** | **0.02** | **<.001** |
| Location (level 1) | - | - | - |  | **0.91**  **(0.88, 0.95)** | **0.02** | **<.001** |
| Recent Nicotine Use (level 1) | - | - | - |  | 1.05  (1.01, 1.10) | 0.02 | .02 |
| Weekend (level 1) | **1.53**  **(1.13, 2.07)** | **0.15** | **.007** |  | - | - | - |
| Minority Stress (level 2) | 0.31  (0.05, 2.04) | 0.94 | .22 |  | **8.24**  **(2.33, 29.1)** | **0.63** | **.001** |
| Social Motives | 0.96  (0.69, 1.34) | 0.17 | .78 |  | 0.96  (0.81, 1.15) | 0.09 | .67 |
| Social Motives x  Minority Stress | 1.19  (0.77, 1.83) | 0.22 | .43 |  | 0.96  (0.92, 1.00) | 0.02 | .07 |
| *Self-Enhancement Motives Model* |  |  |  |  |  |  |  |
| Intercept | 0.90  (0.64, 1.27) | 0.17 | .54 |  | 2.99  (2.52, 3.56) | 0.09 | **<.001** |
| Minority Stress (level 1) | 0.81  (0.51, 1.27) | 0.23 | .35 |  | 1.14  (1.08, 1.19) | 0.02 | **<.001** |
| Location (level 1) | - | - | - |  | 0.91  (0.88, 0.95) | 0.02 | **<.001** |
| Recent Nicotine Use (level 1) | - | - | - |  | 1.05  (1.01, 1.10) | 0.02 | .02 |
| Weekend (level 1) | **1.52**  **(1.12, 2.05)** | **0.15** | **.008** |  | - | - | - |
| Minority Stress (level 2) | 0.30  (0.05, 1.93) | 0.94 | .20 |  | **6.75**  **(1.92, 23.67)** | **0.63** | **.003** |
| Self-Enhancement Motives | 1.02  (0.73, 1.44) | 0.17 | .47 |  | 1.19  (0.99, 1.42) | 0.09 | .07 |
| Self-Enhancement Motives x  Minority Stress | 1.24  (0.77, 2.00) | 0.24 | .37 |  | 0.97  (0.93, 1.01) | 0.02 | .16 |
| *Boredom Relief Motives Model* |  |  |  |  |  |  |  |
| Intercept | 0.92  (0.66, 1.28) | 0.17 | .62 |  | **2.99**  **(2.51, 3.57)** | **0.09** | **<.001** |
| Minority Stress (level 1) | 0.79  (0.50, 1.25) | 0.23 | .31 |  | **1.14**  **(1.08, 1.19)** | **0.02** | **<.001** |
| Location (level 1) | - | - | - |  | **0.91**  **(0.88, 0.95)** | **0.02** | **<.001** |
| Recent Nicotine Use (level 1) | - | - | - |  | **1.05**  **(1.01, 1.10)** | **0.02** | **.01** |
| Weekend (level 1) | **1.51**  **(1.12, 2.05)** | **0.15** | **.008** |  | - | - | - |
| Minority Stress (level 2) | 0.27  (0.04, 1.71) | 0.92 | .16 |  | **7.56**  **(2.07, 27.57)** | **0.65** | **.003** |
| Boredom Relief Motives | 1.28  (0.99, 1.66) | 0.13 | .04 |  | 1.05  (0.91, 1.22) | 0.07 | .51 |
| Boredom Relief Motives x  Minority Stress | 1.10  (0.77, 1.57) | 0.18 | .59 |  | 0.97  (0.93, 1.00) | 0.02 | .05 |
| *LGBTQ-Specific Motives Model* |  |  |  |  |  |  |  |
| Intercept | 0.91  (0.64, 1.28) | 0.17 | .57 |  | **2.98**  **(2.51, 3.55)** | **0.09** | **<.001** |
| Minority Stress (level 1) | 0.87  (0.54, 1.39) | 0.23 | .55 |  | **1.16**  **(1.10, 1.22)** | **0.03** | **<.001** |
| Location (level 1) | - | - | - |  | **0.91**  **(0.88, 0.95)** | **0.02** | **<.001** |
| Recent Nicotine Use (level 1) | - | - | - |  | **1.05**  **(1.01, 1.10)** | **0.02** | **.01** |
| Weekend (level 1) | **1.51**  **(1.11, 2.05)** | **0.15** | **.009** |  | **-** | **-** | **-** |
| Minority Stress (level 2) | 0.24  (0.04, 1.60) | 0.94 | .14 |  | **6.26**  **(1.74, 22.52)** | **0.64** | **.006** |
| LGBTQ-Specific Stress Reduction Motives | 1.10  (0.84, 1.44) | 0.14 | .10 |  | 1.15  (1.00, 1.33) | 0.07 | .13 |
| LGBTQ-Specific Social Motives | 1.04  (0.78, 1.38) | 0.14 | .50 |  | 0.94  (0.81, 1.10) | 0.08 | .53 |
| LGBTQ-Specific Stress Reduction Motives x Minority Stress | 1.39  (0.91, 2.15) | 0.22 | .13 |  | **0.95**  **(0.91, 0.98)** | **0.02** | **.005** |
| LGBTQ-Specific Social Motives x Minority Stress | 1.15  (0.75, 1.76) | 0.22 | .53 |  | 1.02  (0.98, 1.06) | 0.02 | .35 |
| *Note:* Adjusted odds ratio (AOR), incidence rate ratio (IRR), and 95% confidence interval (CI). Covariate reference groups: location is elsewhere (i.e., not home/dorm), recent nicotine use is no, and weekend is weekday. All motives are between-participants (level 2). Significant results (*p* < .01) are bolded.  ^1^These analyses were conducted in the day-level sample (*N* = 75).  ^2^These analyses were conducted in the momentary sample (*N* = 83). | | | | | | | |

**Figure S1.**

*Sensitivity Analysis Interaction Plot for Minority Stress and Stress Reduction Nicotine Use Motives Predicting Nicotine Craving*

***Note.*** Craving was estimated using reference groups for location (not at home/dorm) and recent nicotine use (none).

**Figure S2**

*Sensitivity Analysis Interaction Plot for Minority Stress and LGBTQ-Specific Stress Reduction Nicotine Use Motives Predicting Nicotine Craving*

***Note.*** Craving was estimated using reference groups for location (not at home/dorm), recent nicotine use (none), and average LGBTQ-specific social nicotine motives.

| Table S2 | | | | | | | |
| --- | --- | --- | --- | --- | --- | --- | --- |
| Sensitivity Analysis – Multilevel Models Examining Day-Level Associations Between Minority Stress and Odds of Nicotine Use | | | | | | | |
| **Variable** | **Nicotine Use Full Model^1^** | | |  | **Nicotine Use Separate Models^2^** | | |
|  | ***AOR***  **(95% CI)** | ***SE*** | ***p*** |  | ***AOR***  **(95% CI)** | ***SE*** | ***p*** |
| *Stress Reduction Motives Model* |  |  |  |  |  |  |  |
| Intercept | **4.14**  **(2.71, 6.33)** | **0.21** | **<.001** |  | **4.09**  **(2.70, 6.19)** | **0.21** | **<.0001** |
| Minority Stress (level 1) | **1.62 (1.15, 2.26)** | **0.17** | **.006** |  | **1.62**  **(1.16, 2.25)** | **0.17** | **.005** |
| Weekend (level 1) | **1.60 (1.24, 2.06)** | **0.13** | **.0005** |  | **1.59**  **(1.23, 2.04)** | **0.13** | **.0005** |
| Minority Stress (level 2) | 4.74 (0.48, 46.51) | 1.15 | .18 |  | 5.41  (0.61, 48.17) | 1.1 | .13 |
| Stress Reduction Motives | 1.21 (0.75, 1.97) | 0.24 | .20 |  | 1.41  (0.94, 2.11) | 0.2 | .048 |
| Stress Reduction Motives x  Minority Stress | 1.29 (0.88, 1.88) | 0.19 | .19 |  | 1.15  (0.85, 1.55) | 0.16 | .38 |
| *Social Motives Model* |  |  |  |  |  |  |  |
| Intercept |  |  |  |  | **4.13**  **(2.70, 6.32)** | **0.21** | **<.0001** |
| Minority Stress (level 1) |  |  |  |  | **1.60**  **(1.15, 2.21)** | **0.16** | **.006** |
| Weekend (level 1) |  |  |  |  | **1.59**  **(1.24, 2.06)** | **0.13** | **.0005** |
| Minority Stress (level 2) |  |  |  |  | 11.30  (1.35, 94.45) | 1.07 | 0.03 |
| Social Motives | 0.88  (0.53, 1.45) | 0.25 | .37 |  | 1.11  (0.73, 1.69) | 0.21 | 0.97 |
| Social Motives x  Minority Stress | 0.82  (0.54, 1.25) | 0.21 | .35 |  | 0.80  (0.57, 1.11) | 0.17 | 0.19 |
| *Self-Enhancement Motives Model* |  |  |  |  |  |  |  |
| Intercept |  |  |  |  | **4.18**  **(2.74, 6.39)** | **0.21** | **<.0001** |
| Minority Stress (level 1) |  |  |  |  | **1.56**  **(1.12, 2.16)** | **0.17** | **.009** |
| Weekend (level 1) |  |  |  |  | **1.60**  **(1.24, 2.06)** | **0.13** | **.0005** |
| Minority Stress (level 2) |  |  |  |  | 10.17  (1.22, 85) | 1.07 | .03 |
| Self-Enhancement Motives | 1.19  (0.69, 2.08) | 0.28 | .81 |  | 1.32  (0.84, 2.06) | 0.22 | .40 |
| Self-Enhancement Motives x  Minority Stress | 0.80  (0.49, 1.31) | 0.25 | .37 |  | 0.84  (0.59, 1.19) | 0.18 | .33 |
| *Boredom Relief Motives Model* |  |  |  |  |  |  |  |
| Intercept |  |  |  |  | **4.17**  **(2.75, 6.34)** | **0.21** | **<.0001** |
| Minority Stress (level 1) |  |  |  |  | **1.60**  **(1.15, 2.22)** | **0.17** | **.006** |
| Weekend (level 1) |  |  |  |  | **1.59**  **(1.23, 2.05)** | **0.13** | **.0005** |
| Minority Stress (level 2) |  |  |  |  | 8.74  (1.05, 72.65) | 1.06 | .045 |
| Boredom Relief Motives | 1.25  (0.84, 1.88) | 0.20 | .22 |  | 1.33  (0.95, 1.87) | 0.17 | .08 |
| Boredom Relief Motives x  Minority Stress | 1.05  (0.76, 1.45) | 0.16 | .75 |  | 1.03  (0.79, 1.35) | 0.14 | .82 |
| *LGBTQ-Specific Motives Model* |  |  |  |  |  |  |  |
| Intercept |  |  |  |  | **4.04**  **(2.65, 6.17)** | **0.21** | **<.001** |
| Minority Stress (level 1) |  |  |  |  | **1.70**  **(1.21, 2.38)** | **0.17** | **.003** |
| Weekend (level 1) |  |  |  |  | **1.59**  **(1.23, 2.05)** | **0.13** | **.0005** |
| Minority Stress (level 2) |  |  |  |  | 6.61  (0.75, 58.29) | 1.09 | .09 |
| LGBTQ-Specific Stress Reduction Motives |  |  |  |  | 1.19  (0.84, 1.68) | .17 | .07 |
| LGBTQ-Specific Social Motives |  |  |  |  | 1.03  (0.72, 1.49) | .18 | .95 |
| LGBTQ-Specific Stress Reduction Motives x Minority Stress |  |  |  |  | 1.36  (1.01, 1.85) | .15 | .05 |
| LGBTQ-Specific Social Motives x Minority Stress |  |  |  |  | 0.95  (0.70, 1.3) | .16 | .77 |
| *Note:* Adjusted odds ratio (AOR) and 95% confidence interval (CI). Covariate reference groups: location is elsewhere (i.e., not home/dorm), recent nicotine use is no, and weekend is weekday. All motives are between-participants (level 2). Significant results (p < .01 ) are bolded.  ^1^All variables were included simultaneously in the full model except LGBTQ-specific motives (*N* = 83).  ^2^ Nicotine use motives and its interactions were included in separate models (*N* = 83). | | | | | | | |

| **Table S3** | | | | | | | |
| --- | --- | --- | --- | --- | --- | --- | --- |
| Sensitivity Analysis – Multilevel Models Predicting Odds of Day-Level Nicotine Use and Momentary Nicotine Craving including Post-hoc Covariates | | | | | | | |
| **Variable** | **Nicotine Use^1^** | | |  | **Nicotine Craving^2^** | | |
|  | ***AOR***  **(95% CI)** | ***SE*** | ***p*** |  | ***IRR***  **(95% CI)** | ***SE*** | ***p*** |
| Intercept | 0.92  (0.65, 1.29) | 0.17 | 0.61 |  | **3.00**  **(2.54, 3.54)** | **.08** | **<.001** |
| *Level 1 (Within-Participants)* |  |  |  |  |  |  |  |
| Minority Stress | 0.80  (0.50, 1.27) | 0.23 | 0.34 |  | **1.15**  **(1.09, 1.21)** | **0.03** | **.04** |
| Location | **-** | **-** | **-** |  | **0.91**  **(0.88, 0.95)** | **0.02** | **<.001** |
| Recent Nicotine Use | **-** | **-** | **-** |  | **1.05**  **(1.01, 1.10)** | **0.02** | **.02** |
| Weekend | **1.52**  **(1.12, 2.07)** | **0.15** | **.008** |  | - | - | - |
| *Level 2 (Between-Participants)* |  |  |  |  |  |  |  |
| Stress Reduction Motives | 1.13  (0.78, 1.64) | 0.19 | .18 |  | 1.11  (0.92, 1.37) | 0.10 | .42 |
| Social Motives | 0.89  (0.60, 1.32) | 0.20 | .88 |  | 0.83  (0.68, 1.02) | 0.10 | .05 |
| Self-Enhancement Motives | 0.94  (0.62, 1.44) | 0.21 | .77 |  | **1.24**  **(1.00, 1.54)** | **0.11** | **.04** |
| Boredom Relief Motives | 1.30  (0.94, 1.81) | 0.17 | .18 |  | 0.98  (0.83, 1.16) | 0.08 | .79 |
| Minority Stress | 0.18  (0.02, 1.33) | 1.00 | .09 |  | **3.80**  **(1.05, 13.80)** | **0.65** | **.04** |
| Social Anxiety Symptoms | 0.99  (0.96, 1.01) | 0.01 | .37 |  | 1.00  (0.99, 1.02) | 0.01 | .51 |
| Depression Symptoms | 1.02  (0.98, 1.06) | 0.02 | .39 |  | 1.01  (0.99, 1.03) | 0.01 | .39 |
| *Cross-Level Interactions* |  |  |  |  |  |  |  |
| Stress Reduction Motives x Minority Stress | 1.37  (0.81, 2.33) | 0.27 | .24 |  | **0.94**  **(0.88, 0.99)** | **0.03** | **.03** |
| Social Motives x  Minority Stress | 1.18  (0.68, 2.06) | 0.28 | .55 |  | 0.96  (0.90, 1.03) | 0.03 | .22 |
| Self-Enhancement Motives x Minority Stress | 0.97  (0.49, 1.92) | 0.35 | .93 |  | 1.03  (0.96, 1.10) | 0.03 | .42 |
| Boredom Relief Motives x Minority Stress | 0.95  (0.63, 1.44) | 0.21 | .82 |  | 0.99  (0.95, 1.04) | 0.02 | .71 |
| *Note:* Adjusted odds ratio (AOR), incidence rate ratio (IRR), and 95% confidence interval (CI). Covariate reference groups: location is elsewhere (i.e., not home/dorm), recent nicotine use is no, and weekend is weekday. Social anxiety and depression symptoms were added as post-hoc covariates. Significant results are bolded.  ^1^These analyses were conducted in the day-level sample (*N* = 75).  ^2^These analyses were conducted in the momentary sample (*N* = 83). | | | | | | | |
